# Identification and Characterization of New *Alu* Element Insertion in the *BRCA1* Exon 14 Associated with Hereditary Breast and Ovarian Cancer

**DOI:** 10.3390/genes12111736

**Published:** 2021-10-29

**Authors:** Ahmed Bouras, Melanie Leone, Valerie Bonadona, Marine Lebrun, Alain Calender, Nadia Boutry-Kryza

**Affiliations:** 1 Department of Molecular and Medical Genetics, Hospices Civils de Lyon, University Hospital, 69500 Bron, France; melanie.leone@lyon.unicancer.fr (M.L.); alain.calender@chu-lyon.fr (A.C.); nadia.boutry-kryza@lyon.unicancer.fr (N.B.-K.); 2 Unit of Prevention and Genetic Epidemiology, UMR CNRS 5558, Centre Léon Bérard, 69008 Lyon, France; valerie.bonadona@lyon.unicancer.fr; 3 Department of Genetics, Saint Etienne University Hospital, 42270 Saint Priez en Jarez, France; marine.lebrun@chu-st-etienne.fr

**Keywords:** hereditary breast and ovarian cancer, next-generation sequencing, *BRCA1*, *Alu*Yb8, retrotransposon

## Abstract

Hereditary breast and ovarian cancer syndrome (HBOC) is an autosomal dominant cancer predisposition syndrome characterized by an increased risk of breast and ovarian cancers. Germline pathogenic variants in *BRCA1* are found in about 7–10% of all familial breast cancers and 10% of ovarian cancers. *Alu* elements are the most abundant mobile DNA element in the human genome and are known to affect the human genome by different mechanisms leading to human disease. We report here the detection, by next-generation sequencing (NGS) analysis coupled with a suitable bioinformatics pipeline, of an *Alu*Yb8 element in exon 14 of the *BRCA1* gene in a family with HBOC history first classified as BRCA-negative by Sanger sequencing and first NGS analysis. The c.4475_c.4476ins*Alu*Yb8 mutation impacts splicing and induces the skipping of exon 14. As a result, the produced mRNA contains a premature stop, leading to the production of a short and likely non-functional protein (pAla1453Glyfs*10). Overall, our study allowed us to identify a novel pathogenic variant in *BRCA1* and showed the importance of bioinformatics tool improvement and versioning.

## 1. Introduction

Hereditary breast and ovarian cancer syndrome (HBOC) is an autosomal dominant cancer predisposition syndrome characterized by an increased risk of breast and ovarian cancers [1]. The breast cancer associated genes *BRCA1* (OMIM #113705) and *BRCA2* (OMIM #612555) are the most well-known breast and ovarian cancer susceptibility genes with a lifetime cancer risk in carriers of pathogenic variants estimated at 60–80% for breast cancer and 20–40% for ovarian cancer [2]. Germline pathogenic variants in *BRCA1* occur in 7–10% of familial breast cancers and 10% of ovarian cancers [3,4].

Retrotransposons (REs) are genetic mobile elements that can “jump” by retrotransposition and are believed to insert (randomly) in the host genome through an RNA intermediate mechanism. REs are subdivided into different groups based on their structures and phylogenetic origins. The two main groups are long terminal repeats (LTR) and non-LTR RE. The non-LTR REs include long interspersed elements (LINEs) and short interspersed elements (SINEs), which represent approximately 30% of the human genome. *Alu* elements are the most abundant SINEs with a typical length of ~300 base pairs (bp). *Alu* elements have no coding capacity and are required to amplify the retrotransposition molecular machinery encoded by LINE-1 (L1). *Alu* insertion into critical genomic regions could be pathogenic, resulting in many diseases by disrupting gene transcription, splicing, and/or translation [5]. To date, more than 100 *Alu* insertions causing Mendelian diseases have been reported, several of which have been detected in genes predisposing for cancer, such as *BRCA2*, *MLH1*, and *APC* [6,7,8]. The most studied *Alu* insertion in HBOC syndrome is the c.156_157ins*Alu*, located in exon 3 of *BRCA2*. This insertion has been shown to cause a full exon 3 skipping of *BRCA2*. The detection of *Alu* insertion is difficult by sanger sequencing and often requires specific PCR conditions [9]. Recently, next generation sequencing (NGS) technologies with high coverage uniformity throughout the entire target region has emerged as a powerful tool to detect *Alu* insertion [10,11]. SOPHiA DDM^®^ platform incorporates an original bioinformatics module that is able to detect some complex variants such as long Indels, CNVs, and *Alu* insertions from NGS data generated by SOPHiA Genetics’ Hereditary Cancer Solution (HCS) kit.

Here, we report the molecular characterization of a novel exonic *Alu* element insertion in *BRCA1*, identified with an NGS based multigene panel in a HBOC family, which was undetectable by Sanger sequencing and first-line NGS analysis. 

## 2. Materials and Methods

### 2.1. Subjects

All subjects in this study were tested after giving informed consent.

**Figure 1:**
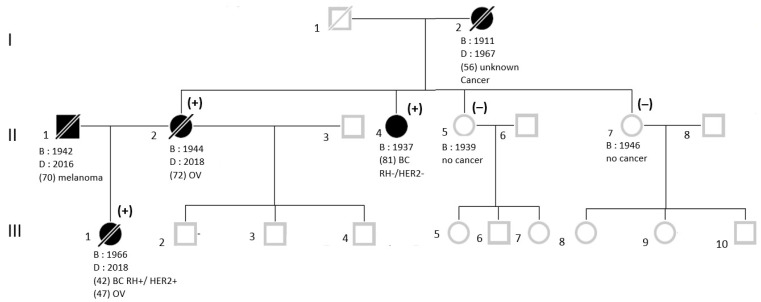
Pedigree of the family assessed for HBOC gene screening. (+): Subject carrying the *BRCA1* c.4475_4476ins*Alu* insertion, (−): Subject not carrying the *Alu* insertion. BC: breast cancer; OV: ovarian cancer; RH: hormone receptors; HER2: human epidermal growth factor receptor 2.

Three probands were analyzed in this family; 

Proband III-1 (Figure 1) developed a left breast cancer at age 42 years and a high-grade serous ovarian cancer at 47. *BRCA1/BRCA2* Sanger sequencing, performed in 2015, did not show any pathogenic variant. The second proband II-2 developed an ovarian cancer at 72. Genetic screening of *BRCA1, BRCA2*, and *PALB2* by Hereditary Cancer Solution (HCS) in 2017 was negative. More recently, a third subject (II-4) developed a left triple negative breast cancer at the age of 81. In order to access antiPARP therapy, this patient was tested by (HCS) kit in 2019.

Total genomic DNA was extracted from blood samples using the automated procedure implemented on the STARlet platform (Hamilton Company, Reno, NV, USA).

### 2.2. NGS Analysis

The DNA of patients II-2 and II-4 was processed by the Hereditary Cancer Solution (HCS) kit (SOPHiA GENETICS, Saint-Sulpice, Switzerland) as described previously [12]. A total of 26 genes are analyzed using the NGS method (*ATM, APC, BARD1, BRCA1, BRCA2, BRIP1, CDH1, CHEK2, EPCAM, FAM175A, MLH1, MRE11A, MSH2, MSH6, MUTYH, NBN, PALB2, PIK3CA, PMS2, PMS2CL, PTEN, RAD50, RAD51C, RAD51D, STK11, TP53,* and *XRCC2*). Bioinformatic data processing for patient II-2 was performed using v4.5.1 of the Sophia DDM software (Saint-Sulpice, Switzerland). A more recent version of the bioinformatic pipeline (v5.4.0) (Saint-Sulpice, Switzerland) was used for data processing for patient II-4 in 2019. 

### 2.3. Characterization for the Alu Insertion

Characterization of the c.4475_4476ins*Alu* mutation, located in exon 14, was performed using two independent PCRs. The first one was performed using forward primer F1 5′- CAGAATCCAGAAGGCCTTTC-3′ located in exon 14 and reverse primer R1 5’-GTGTATAAATGCCTGTATGCA-3’ located in intron 14 in order to flank the *Alu* element. KB extender (3%) (Thermo Fisher Scientific, Invitrogen, Villebon sur Yvette, France) was added to the mixture. PCR conditions were denaturation at 95 °C for 5 min for 35 cycles (denaturation 95 °C for 30 s, annealing 52 °C for 30 s, extension 72 °C for 120 s) followed by final extension at 72°C for 5 min.

The second one is a specific PCR using a forward primer F2 5’-CTAACCTGAATTATCACTATCA -3’ located in intron 13 and a reverse primer R2 5’-GGACTGCAGTGGCGCAAT-3’ specific to the *Alu* insertion. PCR conditions were denaturation at 95 °C for 5 min for 35 cycles (denaturation 95 °C for 30 s, hybridization 58 °C for 30 s, extension 72°C for 120 s) followed by final extension at 72 °C for 5 min.

### 2.4. RT-PCR Analysis

RNA from patient II-4 was extracted from the PAXgene Blood RNA Kit (PreAnalytiX, Qiagen, Valencia, CA, USA) and used for cDNA synthesis (Superscript III First-Strand Synthesis SuperMix, Invitrogen, Villebon sur Yvette, France). PCR was done using a Platinum™ Taq DNA Polymerase (Invitrogen) and primers covering a region between exon 13 and 15 (forward :CAGCAGGAAATGGCTGAACT-3’ and reverse: 5’-GAGCTCCTCTTGAGATGGGT-3’) with the following reaction conditions: 95°C for 4 min, initial denaturation, 14 cycles of 1 min at 95°C, 1 min at 62°C with an increase of 0.5°C every PCR cycle, 2 min at 72°C, 25 cycles of 1 min at 95°C, 1 min at 55°C, 2 min at 72°C followed by 7 min at 72°C for subsequent Sanger sequencing. Control RNA was extracted from HBOC patients without the c.4475_4476ins*Alu*Yb8 insertion in *BRCA1*. The detailed protocol is available on request. The recommendations of the French Unicancer genetic Group were followed for the interpretation of the results [13].

## 3. Results

### 3.1. NGS Sequencing

Analysis of III-1 and II-2 were first negative for the BRCA1/BRCA2 pathogenic variant with the Sanger analysis and NGS analysis. A third proband (II-4) was tested in order to access PARP therapy with the CE-IVD Hereditary Cancer Solution (HCS) assay by SOPHiA Genetics. NM_007294.4 was used for the nomenclature description of the *BRCA1* gene. The result revealed the presence of an insertion of 202 bp at c.4475_c.4476 in *BRCA1* exon 14 with a variant allele frequency of 22%. Evidence of the *Alu* sequence was revealed by Integrative Genomics Viewer (IGV), which showed the presence of chimeric soft-clipped reads coming from the *Alu* element, some of which are carriers of the polyT stretch (Figure S1).

### 3.2. Alu Sequence Characterization

**Figure 2:**
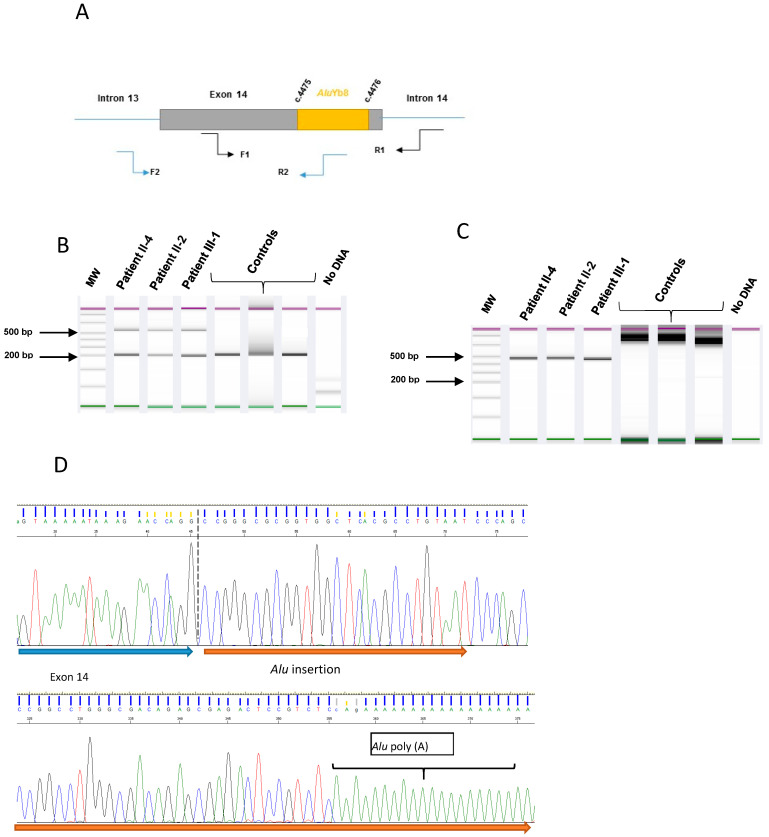
*Alu* insertion in exon 14 of the *BRCA1* gene in the three HBOC patients compared with three controls. (**A**) Schematic illustration of *Alu* insertion and PCR primer position. PCR F1-R1: *Alu* flanked PCR, PCR F2-R2: Specific *Alu* PCR. (**B**) Automated gel electrophoresis using the TapeStation detection system of the *Alu* flanking PCR products—a supplementary 524 bp band was shown in patients and not in controls. (**C**) Specific *Alu* PCR: the expected 450 bp fragment from patient samples and no specific bands observed from control samples. (**D**) Identification of the insertion by Sanger sequencing (forward).

To better characterize the sequence of the *Alu* insertion, two PCRs were performed. The first PCR flanking the *Alu* element revealed the expected normal band at 205 bp and a supplementary band at 524 bp. The specific *Alu* element PCR revealed the expected 450 bp band in the patient samples and none in the controls. Sanger sequencing of PCR products revealed an insertion of an *Alu* RE consisting of 319 nucleotides followed by a poly A stretch of 32A (Figure 2). Sequence blast on Dfam database (Dfam 3.3 software; University of Montana, Missoula, MT, USA) showed that the *Alu* element belonged to the *Alu*Yb8 subfamily [14].

The c.4475_4476ins*Alu*Yb8 mutation was detected in the DNA samples from III-1 and II-2 but not from the unaffected siblings (II-5 and II-6, Figure 1).

### 3.3. RNA Analysis

**Figure 3:**
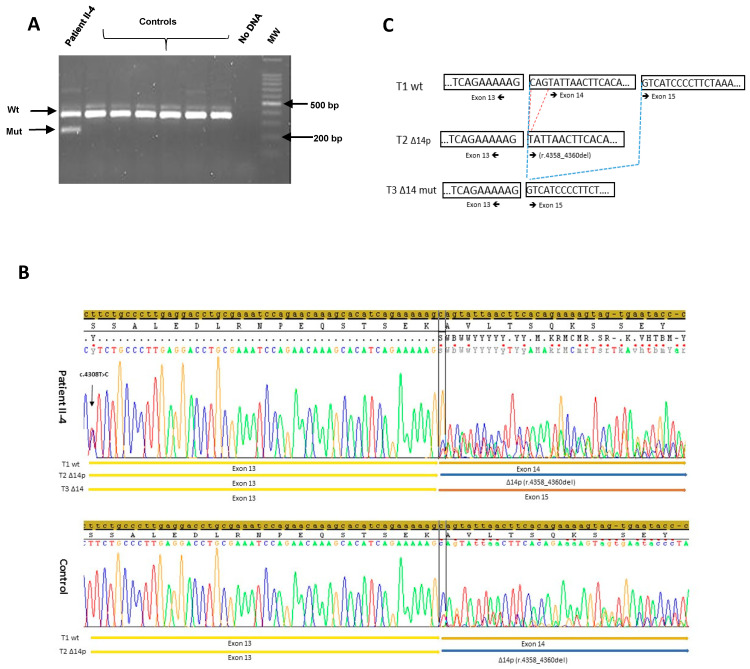
cDNA expression of the allele harboring the *Alu* insertion. (**A**) Agarose gel electrophoresis of the RT-PCR performed with mRNA obtained from patient II-4 and non-carrier controls. The exon 14 skipping (Δ14) and wild type (WT) alleles are predicted to produce bands of 236 and 362 bp, respectively. (**B**) Sanger sequencing of the RT-PCR product of the sample from patient and non-carrier control. Physiological transcripts “T1 wt” and “T2 Δ14p” are present in both patient and non-carrier control and the aberrant transcript that led to exon 14 skipping (T3 Δ14) is present only in patient II-4 sample. The alternative spliced transcript (Δ14p: r.4358_4360del) is observed physiologically at a rate of 40% [15]. The c.4308T > C single-nucleotide polymorphism present in the patient II-4 sample indicated that both alleles were amplified. (**C**) Schematic representation of the three transcripts observed in the patient II-4 sample.

RNA analysis was conducted for ID II-4. RT-PCR was performed by a single primer set designed to amplify the BRCA1 cDNA sequence flanking exon 14. We obtained two bands with different seizes after gel electrophoresis: a band at the expected size of the amplified fragment from the wild type allele and a short band of about 236 bp. Sequence analysis of the short fragment evidenced abnormal splicing with total skipping of exon 14. This abnormal transcript leads to a frameshift and a premature stop codon, allowing us to consider the *Alu*Yb8 insertion as a deleterious variant for the protein functional structure. (Figure 3).

## 4. Discussion

In this study, a SOPHiA Genetics HCS gene panel allowed us to detect a novel exonic c.4475_4476ins*Alu*Yb8 insertion in *BRCA1* in a family with HBOC history first classified as BRCA-negative by Sanger sequencing. A further characterization of this variant at the RNA level identified the presence of an abnormal transcript that leads to a frameshift and a premature stop codon r.4358_4484del p.(Ala1453Glyfs*10). An analysis of other family members showed the segregation of this variant with ovarian and breast cancers, thus suggesting its implication in the HBOC syndrome reported in this family. The study presented here provides the evidence that genetic exploration must be continued in families with a significant history of cancer for which no pathogenic variant has been found by routine genetic tests. In addition to its etiological diagnostic value, this analysis was of theranostic value because it indicated a personalized therapeutic strategy using antiPARP [16].

To our knowledge, this is the first report of an *Alu* insertion in exon 14 of *BRCA1*. Several cases of *Alu* insertion in cancer predisposition genes have been reported in the literature, most of which are pathogenic due to their effect on splicing [7,9,10]. Exons 11 of *BRCA1* and *BRCA2* showed a higher *Alu* insertion ratio than other cancer predisposition genes’ exons, which may be explained by their large sequence sizes [8].

NGS technology, coupled with a suitable bioinformatics pipeline, have emerged as a powerful tool to detect these types of variants since PCR-based methods with standard conditions are not always able to amplify *Alu* insertion alleles due to their large size and the presence of a terminal poly (A) sequence [8]. In this case, the number of reads covering *Alu* insertion is relatively low (22%) compared to the expected rate of heterozygosity. The possibility of mosaicism has been excluded due to the autosomic transmission of this family. This lower rate could then be explained by a loss of part of the chimeric reads during the capture and alignment process and may be the cause of undiagnosed HBOC families. In addition, it is possible that some Res’ insertions (i.e., large L1 or SVA insertions) are difficult to detect using massively parallel short-read sequencers. Fortunately, long read sequencing technologies that may span the entire length of full transposons are now available to us [17].

Interestingly, Patient II-2 benefited from the same CE-IVD Sophia Kit in 2017. No pathogenic variant was found at the time and the *Alu* insertion was not detected. The reprocessing of the 2017 ID II-2 FASTQ data with the latest version of the bioinformatics pipeline of Sophia Genetics revealed the presence of this *Alu* insertion. This could be explained by an improvement in the bioinformatics pipeline by Sophia Genetics between 2017 and 2019 to better detect *Alu* insertions with increased sensitivity. Therefore, laboratories must always improve bioinformatics pipelines and set up a quality management system (i.e., ISO15189) for better software version traceability in order to detect such challenging variants.

## Data Availability

Not applicable.

## Supplementary Materials

The following are available online at https://www.mdpi.com/article/10.3390/genes12111736/s1, Figure S1: Integrative Genomics Viewer (IGV) screen capture showing the alignment of the two *BRCA1* alleles.
