# Supplementary material for: Identification and Characterization of New Alu Element Insertion in the BRCA1 Exon 14 Associated with Hereditary Breast and Ovarian Cancer
Source: Genes (Basel). 2021 Oct 29;12(11):1736. doi: 10.3390/genes12111736 (PMC8623961; doi:10.3390/genes12111736)
Supplement: Supplementary file 1 [file genes-12-01736-s001.zip › genes-1440099-supplementary.pdf]

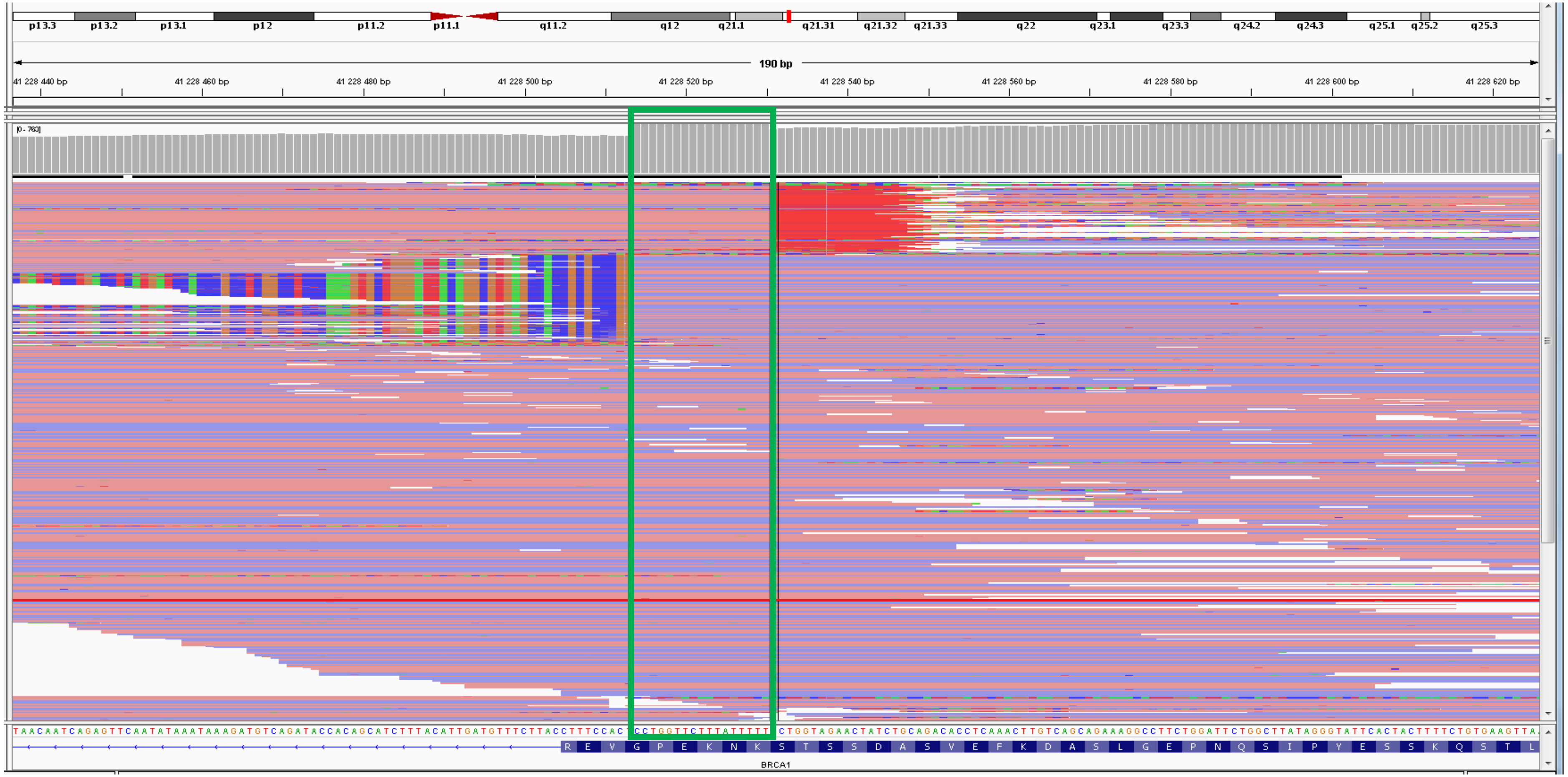

Figure S1. Integrative Genomics Viewer (IGV) screen capture showing the alignment of the two *BRCA1* alleles. The paired-end reads obtained from patient II-4 were aligned to the human genome hg19 in the genomic range: chromosome 17, position 41,228,420 to 41,228,608. Chimeric reads containing the softclipped Poly-T reflecting the 3’ end and the softclipped 5’ start of the *Alu* element are shown (right red stretch and left multicolor stretch). The *Alu* Tandem Duplication Site (TDS) is boxed with green.
